# Four moral models of global health security

**DOI:** 10.1371/journal.pone.0348139

**Published:** 2026-05-06

**Authors:** Frances Charlotte Butcher, Patricia Kingori, Michael Parker

**Affiliations:** The Ethox Centre, Nuffield Department of Population Health, University of Oxford, Oxford, United Kingdom; Thammasat University, THAILAND

## Abstract

Global health security is concerned with the response to infectious disease outbreaks across international borders. However, its precise nature and scope are contested because of lack of agreement about its focus and beneficiaries. Informed by qualitative interviews with thirty-eight professionals working in the field, this article explores views on how the scope and boundaries of global health security should be understood. The findings illustrate the ways in which those working in global health security operate in a complex moral landscape, with a wide variety of definitional boundaries about what global health security means. Our thematic analysis of this contested area suggests that views on global health security can be helpfully understood in terms of four models, each with different conceptual and moral priorities. We label these four moral models as follows: threat model, collective model, reframing model, and humanitarian model. Understanding the field of global health security in terms of these four sometimes competing models provides a helpful way of mapping out and understanding the complex range of agreements and disagreements that arise in relation to epidemics and other global health security events.

## Introduction

When public health efforts to prevent, prepare for, and respond to infectious diseases transcend international borders, it is common to hear discussion about ‘global health security’. Although there is not one accepted meaning of global health security, a commonly used definition is the World Health Organization’s characterization of global public health security as *“the activities required, both proactive and reactive, to minimize vulnerability to acute public health events that endanger the collective health of populations living across geographical regions and international boundaries”* [1]. A proactive activity might include surveillance to identify infectious diseases before they become outbreaks and reactive activity would include measures to control an outbreak identified. Despite global health security becoming an increasingly prominent area of international focus in the twenty-first century, some of the tools of the enterprise, such as border controls and quarantine, originate back to the fourteenth century, when epidemics such as the Black Death led to a focus on unilateral quarantine regulations on European ports [2]. Despite the question of origins, the definition of global health security has become both focused on infectious disease outbreaks across borders [3], and mired in *“ambiguity”* [4] and “*complexity”* [5].

Rushton suggests a key reason why definitions are contested is a concern that the dominant conceptualisation of global health security is often seen as being primarily about the containment of diseases for the protection of Western countries [3]. This concern is prevalent in the securitisation of global health literature, that describes the process by which threats to global health, are socially constructed as a security threat [6]. Here, the securitisation of infectious disease outbreaks is seen to have led to the Global North prioritising their own interests by containing health security threats abroad and/or encouraging strong border controls [3,7–9].

Self-interest has been proposed as an explanation for lack of responses to the West African Ebola epidemic until the first infections in patients in Europe and the US [10]; the border controls imposed in some countries during the 2009 swine influenza pandemic and the 2014 Ebola epidemic that were contrary to WHO recommendations [11–17], and in the mission of the US Agency for International Development to end extreme poverty, primarily in order to advance US security and prosperity [5].

However, self-interest has also been argued to be a useful motivator that *“lubricates the ﬂow of resources”* [18] and motivates countries to invest in tackling (re)emerging infectious diseases [9,19–21]. The argument behind this is to suggest it is in every country’s self-interest to help countries with weaker health security [Tappero et al. in 12].

This association with increased resources may partially explain another trend in arguing for the expansion of global health security in different ways. There have been arguments for improving the global health security contribution to the One Health [22,23], adding a conception of individual health or human security to alter the enterprise to a person-centred focus on access to healthcare and preventive measures [3,12,24–29], implementing global health security in partnership with universal health coverage [12,30–33], and including non-communicable diseases [12,34]. This, in turn, has been subject to critique with Wenham’s [35] analysis of the ‘oversecuritisation of everything’ leading to difficulties distinguishing global health security from other global health efforts.

Despite this rich debate, notable by their absence are perspectives from practitioners of global health security, as well as contributions from bioethics or public health ethics. There are, as a consequence, important empirical, conceptual and normative gaps in the literature around the concept of global health security. There is an empirical gap of how those who work in or outside the global health security paradigm understand the concept. There are conceptual and normative gaps about what global health security should stand for as an enterprise and what boundaries should be drawn. These are important gaps to fill, because increasing understanding of perspectives on this can help those in global health security to navigate its complexity, disagreements, and moral dimensions in order to make progress in protecting persons globally. The research reported in this article aims to fill these gaps by exploring perspectives of those working in global health security about how they understand the concept and the values that underpin it.

## Methods

### Methodology

The data presented in this article were collected as part of a larger study undertaken for [FB]’s doctorate [36]. The overall approach adopted in the research project of which that reported in this paper is a part is that of symbiotic empirical ethics, which aims to integrate empirical data with normative enquiry [37]. Within that context, this article focuses in particular on a key theme from the qualitative analysis.

### Method

Qualitative data collection was undertaken between 11^th^ June 2021–11^th^ April 2022, by means of 38 semi-structured interviews. Interviews were conducted with public health or policy professionals who currently or previously identify as being involved in global health security – broadly understood – and/or working in related areas in the context of acute infectious disease outbreaks (including from public health, government, NGOs, think tanks, and academia). The sampling strategy used a mix of purposive and snowball sampling. The purposive sampling strategy was aimed at identifying and gathering data from a varied range of perspectives, and was informed by a stakeholder mapping exercise to increase diversity in participants by geographical location, sector, and professional background. The study was designed prior to the start of the COVID-19 pandemic, but the interviews were undertaken during it.

Interviewees were contacted primarily by email, with an initial invitation including a participant information sheet, and a reminder email if no response. Of the 63 potential interviewees invited to participate, 38 (60%) chose to participate in the study. Interviews were conducted remotely by FB from two different time zones (Greenwich Mean Time/ and Pacific Standard Time). A semi-structured topic guide was developed with questions on interviewee’s role, definitions of global health security, ethical concerns within global health security and COVID-19. The topic guide was iteratively adapted as the interviews progressed, to reflect how well questions worked and to include ideas brought up unprompted by interviewees that seemed pertinent to research aims.

The decision to cease data collection relied on the theoretical idea of data saturation. In practical terms this meant that a decision was made to conclude data collection at a point when it was judged that the data collected were comprehensive and in depth, and when there appeared to be no new key insights or themes emerging, rather than seeking all data collected to be repetitious [38,39]. Some of the key ideas seemed to reach saturation at an earlier point, such as the idea of boundary setting around the definition of global health security. However, we were keen to ensure this was due to the fact that all the key themes in these areas had been explored, rather than that some kinds of people had been excluded from the study. Therefore further efforts were made to purposively recruit participants with backgrounds that were underrepresented in the sample, including those working in the Global South [38,39]. However, as the sample was expanded, there were few new perspectives gained (see Limitations section). Data collection therefore continued until April 2022 when it was judged that this further recruitment was producing any further new ideas.

The majority of interviewees resided in Europe (47%), North America (24%) or Africa (18%), and were senior career professionals (63%) with a background in health (55%) or public health and epidemiology (47%) (Table 1). A common career path for participants included multiple years of postgraduate training or education to work in healthcare before moving onto careers in areas of public health, global health security, or humanitarian work.

**Table 1:** Interviewee characteristics.

		n	(%)
Employer location^1^	Europe North America Africa Asia Latin America and the Caribbean	18 9 7 2 2	(47) (24) (18) (5) (5)
2022 World Bank income classification^2^	High-income economies	28	(74)
	Upper-middle-income economies	3	(8)
	Lower-middle-income economies	3	(8)
	Low-income economies	4	(11)
Career level	Senior Mid or mid-senior Early or early-mid	24 10 4	(63) (26) (11)
Sector^3^	Non-governmental or non-profit organisation Government or national public health organisation Academia International organisation or partnership Military For profit organisation, consulting or self-employed	16 13 10 7 5 3	(42) (34) (26) (18) (13) (8)
Background^3^	Health professional Public health and epidemiology Policy, law and strategy International development and global health Emergency preparedness and operations Biomedical science and biotechnology Other, including engineering, military, business development, executive, diplomatic	21 18 7 7 4 4 4	(55) (47) (18) (18) (11) (11) (11)
Mean interview length	46 minutes		

^1^Based on regions from UN Geoscheme, and considering country that is primary base for work.

^2^2022 World Bank income classification of country of employment.

^3^Total >38 total interviewees (>100%) as some interviewees define in more than one category.

In the results presented, pseudonyms are used for interviewees, alongside contextual information on working location and current role.

### Data analysis

Audio recordings were transcribed by a professional transcriptionist and uploaded to NVivo V1.6 [40]. The interview transcripts were analysed by FB using thematic analysis, a six stage method for analysing qualitative data in order to identify key themes that explained that data [41,42].

After data familiarization, initial interviews were coded in great detail, with line-to-line coding, to generate a large number of initial codes (50+). These initial codes were then grouped into broader codes that were related through meaning or topic. After the first half of the data was coded, we started the process of collating codes into related groups, to build themes. This involved an iterative process of moving between codes to identify commonalities. These initial themes were discussed and reviewed once all the dataset was coded, and at this point some of the latent themes, that categorise the underlying ideas or assumptions in the data were developed.

To increase rigor, FB, PK, and MP all read and discussed the initial transcripts coded by FB, followed by regular monthly discussion of coding extracts and themes. This allowed for themes and codes to be challenged and analysed at an early stage. In a second coding group of four doctoral students, a further transcript extract was coded by all four members with results compared to increase inter-coder reliability. Reflective notes were written by FB before, during, and after conducting the qualitative study to constitute a form of self-appraisal in research [43]. FB discussed key methodological and analytical decisions with PK and MP.

### Ethics approval and consent

Approval to conduct this study was received from the University of Oxford Medical Sciences Interdivisional Research Ethics Committee (reference R62941/RE001).

Care was taken to ensure interviewees provided informed consent to participate in the study. Interviewees received the information sheet and consent form before the interview, then the study and its risks were discussed at the start of each interview. All interviewees completed a written consent form, except for two: one confirmed consent via email, and the other provided oral consent in line with University of Oxford guidance on oral consent.

## Results

The analysis of these thirty-eight semi structured interviews explores how interviewees define global health security in two key ways. The first is by setting boundaries for what should and should not be included within the concept. This includes for COVID-19 and Ebola, endemic disease, non-communicable diseases and health systems strengthening and deliberate and accidental outbreaks. The second is by raising moral concerns about the nature of global health security, that is, they see the choice of definition as morally significant. By examining these two ways of defining global health security together, our analysis demonstrates that perspectives on global health security can be categorized into four distinct moral models, with different conceptual and moral priorities, which we label as the threat model, the collective model, the reframing model, and the humanitarian model.

### Boundary setting

A key strategy adopted by interviewees when asked to define global health security was to specify or list what issues and diseases were inside or outside the concept, that is, to set boundaries using examples. This was both something explored explicitly in the interview topic guide (for example, prompts on: *“is malaria a global health security problem? Why?/ Why not?”*), but was also something interviewees spontaneously discussed.

#### COVID-19 and Ebola.

There was consensus on only two diseases, COVID-19 and Ebola, that were both seen as global health security problems by all interviewees. This is unsurprising, and reflects the experiences of interviewees in living through, and working to mitigate COVID-19, and many who also worked on previous Ebola outbreaks. It is also consistent with the significant volume of both policy and academic literature framing COVID-19 as a global health security problem [29,44–46].

However, even for the COVID-19 pandemic, one interviewee that noted that the mortality rate of COVID-19 meant that they would not have, prior to the pandemic, “*have been as concerned about”* (Ash, North American, epidemiologist). This gives insight into perspectives from some interviewees that global health security is only concerned with the most serious of infectious diseases – that initially even the virus that went on to cause the most serious pandemic of the century – was not considered sufficiently concerning to constitute a global health security problem.

#### Endemic diseases.

The agreement that COVID-19 and Ebola were global health security problems was in contrast to the differing views of interviewees’ endemic diseases. One interviewee used heavily militaristic language to differentiate between the global health focus on tuberculosis, HIV and non-communicable diseases and the health security focus on outbreaks like Ebola and COVID-19.

*HIV is like a couple that skirmish with your neighbour, where health security is like a war. Because here you have the potential for escalation, you put troops on the border and then that’s it, you know you’ve maintained your defence agenda, but health security is the surprise attack, it’s the…it’s very different.* Berhane (East African, emergency preparedness)

This interviewee’s comments reflected a common thread among interviewees that global health security is about the scale and surprise of the *“attack”* with this level of escalation – of diseases that spread rapidly through large areas. There is also an interesting element in that HIV, one of the original securitised diseases, is now excluded from this paradigm [47]. This is likely because it is now understood – when treated – to be a chronic disease, in contrast to the fearful threat it was when it first began to spread in the 1980s.

This distinction between diseases of global health and those to be understood in terms of global health security differed from the boundaries described by other interviewees, especially with regards to malaria. Malaria was an example used in the interview topic as a potentially interesting boundary case. Whilst some interviewees suggested malaria was a global health security problem because it was a disease moving through a world characterised as a *“global village”*, two other interviewees suggested that malaria is not a global health security problem, but *“it should be”*. Through delineating the difference between their perceived understanding of global health security and their ideas about what ought to be in it, they indicate that the boundaries of global health security are external, and not as they wished them to be.

#### Non communicable diseases and health systems strengthening.

Interviewee perspectives on the role of non-communicable diseases in global health security also varied. Interviewees, for whom this was a new concept introduced by the interviewer, often rejected these as *“global health concerns”* rather than global health security threats, but some interviewees grounded them definitively within the paradigm of global health security.

*I take a very broad perspective…which is that it [global health security] can really be anything that affects our health, that can cross borders…This is a view, for example, that tobacco is a global health security threat...That the actual global international and tobacco companies because of the way that they strategically try to damage health in a way that crosses borders that that is a…global health security threat.* (Eike, North European, health policy).

This helps reveal another quality of global health security threats for some interviewees. As well as urgency and the serious nature of some infectious diseases, global health security can also be about diseases for which a collective response is required to reduce the societal burden of disease caused by commercial actors. Given this perspective, it is unsurprising that many of the interviewees that held this position also argued that health systems strengthening is part of global health security, with one interviewee defining health security as needing *“strong public health”*, *“resilient health systems”*, and *“enabling healthy populations”* (Carey, North European, public health). What it notable is that these perspectives on non-communicable diseases and health systems strengthening were strongly held by interviewees, but seemed to reflect more a position which they advocated for, rather than necessarily being an accepted part of global health security.

#### Deliberate and accidental outbreaks.

The connection between, and inclusion – under the concept of global health security – of both, deliberate outbreaks (the use of a pathogen as a bioweapon) or accidental outbreaks (the accidental release of pathogens from laboratories) was a straightforward link for some interviewees, who discussed these events, one confidently articulating that global health security includes “*the range of problems from deliberate, accidental and natural origins of biological events”* (Blair, North American, health security). This perspective was consistent with the distinction made between natural, accidental and deliberate outbreaks in policy. A policy example here is in the Global Health Security Agenda 2024 Framework, which starts with *“Countries continue to face health security threats posed by infectious diseases, whether naturally occurring, deliberate, or accidental”* [48]*.* However, whilst most interviewees who discussed deliberate and natural causes emphasized the importance of these, some did not consider deliberate or accidental events at all – when their sphere of work was the traditional public health area of natural outbreaks. This meant that, whilst not all interviewees considered these concerns, no interviewee rejected these as part of global health security.

### Moral concerns about global health security

As well as setting boundaries to what is included or excluded in global health security based on different categories of pathogen interviewees’ expressed moral concerns about the enterprise and language of what global health security is. These concerns, which were expressed by many interviewees, are relevant to the meaning of global health security because they are concerns about the enterprise and language of what global health security is. By interviewees expressing these concerns, insights are gained about the moral meaning and standards global health security should have.

A key moral concern was that the security framing led to an “*us and them mentality”* (Alvi, North European, public health). The concern expressed by interviewees was that global health security framing can be ethically problematic because it positions the Global North as the beneficiaries of measures to strengthen health security, while casting the Global South as the supposed source of infectious disease outbreaks. This framing, thus, unjustly prioritises the health of populations in the Global North rather than being truly global. This framing is seen as a wider problem arising out of differences in the value attributed to lives in the Global North versus the Global South, as exemplified by Jo:

*I think there is a darker side to it which is, ‘We care about my health, and I don’t care about your health, and this is about security of my country of me.’ And I think unfortunately that is often how it’s portrayed in, in non-rich world countries that the global health security agenda is actually about protecting people in rich countries.* (Jo, North European, NGO leadership)

Interviewees were also concerned that the issues that global health security represents should be important in and of themselves and should not *need* the security rhetoric to make them important. Robin objects to the need for security as a motivator for decision-makers suggesting “*it would be a better environment if these issues were taken seriously because they are, they are important, not just because they are security threats.”* (Robin, North European, international law).

A key question when looking at the question of moral concerns in global health security, is whether it is the activities in global health security or the framing and the language, or both, that interviewees were objecting to. Some interviewees did find it to be the terminology, rather than the connection between security and health that they found troubling. One interviewee put this very strongly:

*I mean I, I think the, the, the words are flawed and I, I would not use them...And you can’t ignore what words, might mean to us, what they may mean to somebody else either in the emotion of it or even the translation of it, and words really do matter…It’s why I don’t like the phrase, I wouldn’t use it myself, I think it has negative connotations and it’s very divisive.* (Bo, North European, infectious disease)

Thus, for a sub-set of interviewees, even though they acknowledged the link between security and health, the use of the terminology of global health security had such negative connotations as to require that it be rejected. However, in contrast, six interviewees all said global health security was a *“useful”* term because it acknowledged the link between global health and security, and helped bring a wider audience into global health – a perspective that is also seen in the global health security literature [see for example Heymann in 49].

### Four moral models of global health security

**Fig 1:**
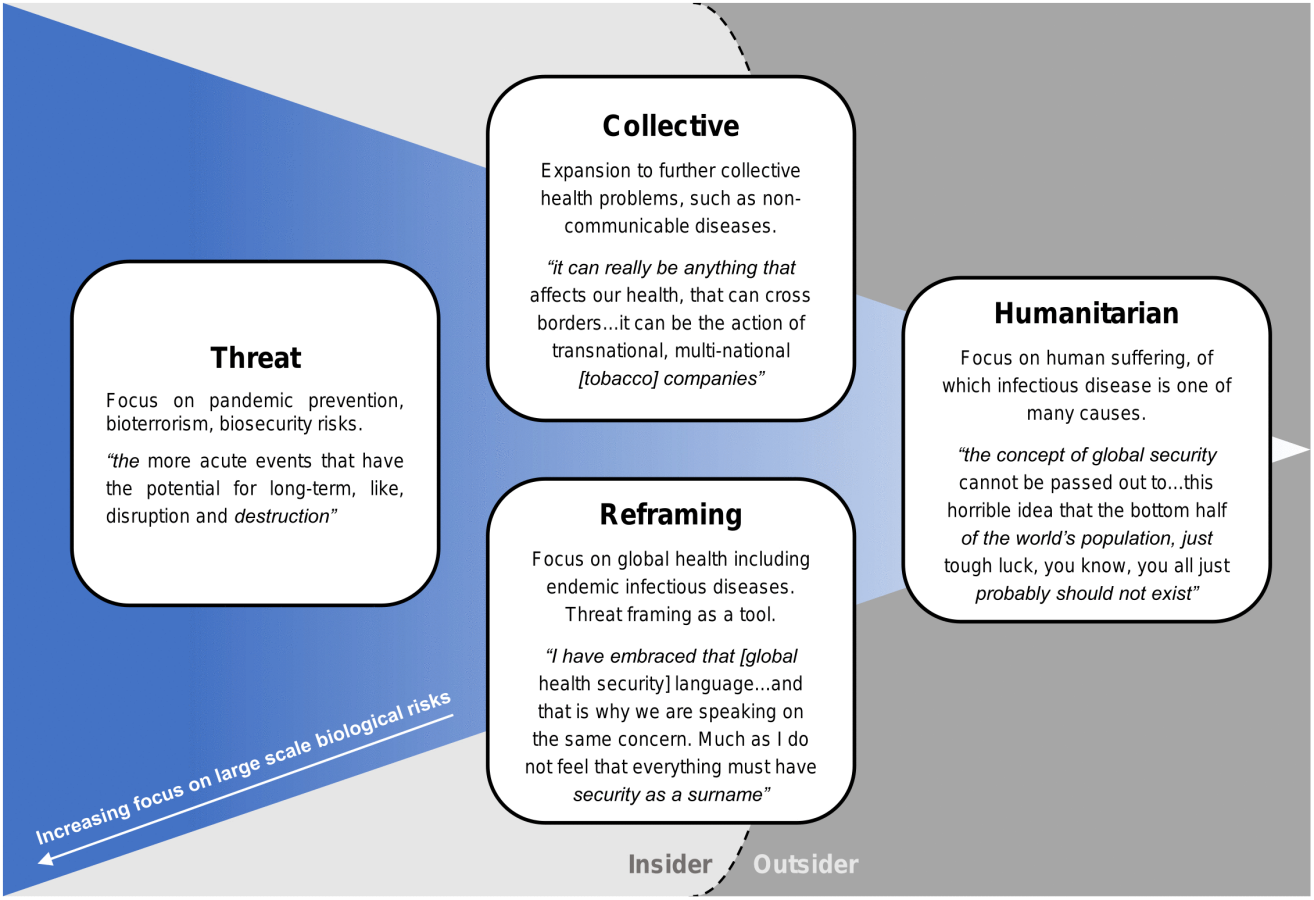
Four moral models of global health security.

In the first part of the results, we have illustrated ways in which global health security can be understood through different boundaries made in relation to pathogen and aetiology of disease, as well as the moral concerns about the enterprise. This section integrates these perspectives to demonstrate that views on global health security can be divided into four different models (Fig 1). These models are grounded in the inductive coding of the interview data, and lead from and build on the earlier analysis on pathogen/disease based boundaries and moral concerns.

These four models, the threat, collective, reframing and humanitarian models, reflect different ways of thinking about global health security. Three of the models are denoted as ‘insider models’ in which the concept of global health security is to some extent accepted, even if this acceptance is contingent on redefining what the term means (Fig 1). One model, the humanitarian model, is reflective of an ‘outsider’ position in which the concept of global health security may be rejected or unknown. Nevertheless it is in included as one of the four models because it is key to understanding the range of perspectives on global health security.

However, it is important to note that these models should not be taken to imply that the view of individual interviewees sit neatly within a single way of thinking about global health security – interviewees often expressed views that fitted into more than one model. These models should be seen, rather, as a typology of perspectives on global health security that interact through the interpretations and practices of individual professionals and groups. For example, a professional who subscribes to multiple models may encounter situations where tensions between these perspectives must be negotiated. This is a dynamic that similarly applies to groups comprising members who adhere to different models.

In a field characterised by difference and disagreement, the value of these models is that they help illustrate and understand the bases of those disagreements. By doing so, these models provide valuable insights that can help global health security professionals understand differing viewpoints and so manage value conflicts more effectively.

#### The threat model.

The threat model is a way of seeing and understanding global health security as an area focusing on large-scale biological events, whether caused by natural outbreaks, accidental release of agents from laboratories, or bioterrorism. It is the model perhaps most closely aligned with the more traditional conceptualisation of global health security, i.e., as the protection of populations from an external threat. Within this model, COVID-19 and Ebola would be included, but non-communicable diseases would not be part of the definition because they are not the events with potential for acute *“disruption and destruction”* (Harley, North American, global health security policy).

This understanding of global health security was characterised by a transition from global health to the different work of global health security, resulting in “*a total paradigm shift for practitioners”* (Berhane, East Africa, emergency preparedness). This finding is important as it confirms that, in this model, global health security is not just a card for health professionals to play to persuade those outside health to provide resources to health, as suggested by Elbe [18], but is seen by some practitioners as a distinct field with a workforce that is different to global health.

This is the model that has been subject to the majority of the moral criticisms of global health security as being perceived to be focused on priorities of the Global North. This is because of the focus on large scale threats that have not yet happened, rather than the current epidemic and endemic priorities that might (but are not necessarily) the focus of some Global South nations. However, this does not imply it has no moral grounding: the moral basis of this model is consequentialist. As Blair states, it is about preventing the large number of deaths and “*suffering in the face of large-scale crises*” and “*preserving the stability of society”* (Blair, North American, Health Security).

#### The collective model.

The collective model arises out of the analysis findings about broadening the boundaries of global health to include more than infectious disease. Specifically, that global health security should include both infectious disease outbreaks, but also other collective health problems that affect populations living across borders, including the importance of paying attention to health systems and non-communicable diseases where these require a collective response. In this model, the language of security is a useful tool to advocate for international work to address these larger problems.

Although this is seen as a collective model, its moral basis is grounded in the concept of individual or human security. It is about instances when collective action is required across borders to enable an individual’s right to access and be in control of their own health. As such, this is a wider view of security as “*the ability of a population to be healthy, to choose to be healthy”* (Vanja, North European, military health).

This reflects a fundamentally different conceptualization of security compared to earlier moral concerns, which predominantly framed global health security as the protection of the Global North from perceived threats emanating from the Global South. By taking an individual understanding of health security, this aims to counter this moral concern, by reframing security to become about the need for collective actions in order to help the individual. In many ways this is an aspiration for what global health security should be, rather than a model of what is it now.

#### The reframing model.

The reframing model is also a response to what are seen as the moral failures of global health security in prioritising the Global North. Rather than proposing a new understanding of global health security focused on individual security as the collective model does, the argument here is about the importance of working within the system to reframe it to have a focus that also includes priority diseases in the Global South, such as malaria, as discussed in boundary setting.

This is a model about improving global health security from the inside (as per Fig 1). It is about the need to engage in the important work of global health security whilst acknowledging some groups may be *“uncomfortable with the securitisation of health”* (Kyrie, North European, public health policy).

In this model, security is not something to aim for, but the way in aims should be framed to achieve their ends of more stable systematic change to health structures. An example of how this functions comes from one interviewee stating “*For those of us working in the space I see the political interest in health security as an opportunity to actually make systemic changes that have a broader health impact”* (Alvi, North European, public health). They give a practical example of this in their work when they interpret a request to set up an Ebola testing laboratory in Sierra Leone, as one that should also meet the pressing local need for being able to conduct hepatitis viral tests.

Whilst this has a lot in common with the collective model in outcomes – aiming for a more sustainable healthcare system – the difference is that the collective model tends to emphasise the larger collective health problems globally, whilst this is focusing on reducing the nationalistic set up that favours the Global North and focusing on aid and sustainability to the Global South.

The moral basis of this model is what we shall refer to as a pragmatic social justice approach. Pragmatic, because the aims are achieved from working within the system, because that is where the resources are. Social justice because this is not about the problem requiring a collective solution but about helping those who are worse off globally. This is summed up eloquently by an interviewee when they suggest “*you just have to be able to titrate your values against how much difference you think you can make* (Teagan, North European, public health).

#### The humanitarian model.

The humanitarian model contrasts with each of the other three models: It is not a version of the original threat model, nor is it collective, nor is it trying to reframe it. In many ways this model should not be considered part of global health security at all, as many of those who may recognise this model would not consider themselves part of the global health security enterprise. However, it is included because it emerged from the analysis as one of the four ways of mapping differences with respect to the idea of global health security.

The key factor in this model is of saving “*lives and alleviate suffering for the most vulnerable” (*Louison, North European, health operations)*.* In some ways, this model has more in common with the threat model than the other two models. This is because it is focused on relieving immediate suffering and thus has a similar urgency and focus on acute events, compared to the sustainability of the collective and reframing models. However, the threat model has a greater emphasis on preventing these biological disasters occurring in the first place. The humanitarian model, by contrast is agnostic about the cause of the event, not distinguishing infectious disease threats from other natural disasters, except by the amount of human suffering caused by these. For example, a threat-oriented approach would view the 2024 H5N1 outbreak in cattle in the United States as a matter of urgent concern because of the human pandemic potential. By contrast, humanitarian actors might assign it lower priority in the absence of widespread human suffering, instead focusing, for example, on the conflict in Ukraine, where acute suffering demands attention even when it is not primarily driven by infectious disease. The distinction, however, is not absolute. Conflicts and natural disasters that generate humanitarian crises often create conditions for infectious disease outbreaks and, in some cases, raise concerns about the potential use of biological weapons, thereby also engaging threat-based reasoning. The divergence therefore lies less in the events themselves than in the primary moral model through which the situation is viewed.

The moral framework for the humanitarian model is all about using resources to maximise the number of lives saved and prevent suffering. It is consequentialist in a different way to the threat model, as it prioritises alleviating current suffering, rather than also future suffering caused by future pandemics, which is central to the threat model. Those who propose these humanitarian ideas seem to engage more explicitly with the ethical concerns about not meeting this need, as Venni does when they discuss the “*horrendous choices about which places you try and put scarce resources in to try and prevent loss of human life”* (Venni, North European, humanitarian).

Security, in the humanitarian model, is not part of the core health work but is a more literal conception based around violence towards persons. For interviewees this related to both security concerns that arise as a consequence of implementing restrictive public health measures such as lockdowns, and concerns about the relationship between humanitarian response and local security services.

## Discussion

The analysis presented here suggests that the way that professionals understand, and act within, global health security can be described in terms of different competing boundaries and moral concerns. The way in which this contestation occurs in the analysis suggests four moral models of global health security. These models offer important insights into the practice of those working in the field.

A criticism of these models might be that they primarily be predictive of different employment sectors – with, for example, the threat and reframing model being associated with government work, and the humanitarian model with humanitarian work. The collective model is harder to categorize but could reflect other sectors such as those working in international organisations, think tanks or academia. This is likely to be somewhat true and reflect the norms of different workplaces. However, it is not that these models represent different interviewees perspectives, but are themes that have emerged from our analysis that suggest four different, sometimes competing, ways of thinking about global health security. Different professionals will likely have some ways of thinking which align with different models in their own understanding of global health security.

### Which model should be used?

An obvious question coming out of the analysis is that if there are four different moral models of global health security, which model should be used? This question in itself presumes the desirability of one widely-agreed understanding of global health security. Whilst this is not necessarily possible, nor desirable, to demarcate global health security so precisely in practice, it is also prudent to consider what are the benefits and risks of expanding global health security beyond the threat model, which is the narrower conception of global health security.

The key ethical argument, that is implied in both the literature [12,26,30–32,50] and the analysis, is that expanding understandings of global health security would solve some of the moral problems inherent in it. The argument here would go that, because moral concerns with the current form of global health security include that it benefits the Global North at the expense of the Global South, expanding it to include more priority diseases of the Global South, as per the reframing model, would make it morally better.

However, this analysis shows why the argument can be problematic. There are interviewees from the Global South who do, to varying extents, adhere to the threat model because of concern with these large-scale biological events. It is also important not to characterise threat model perspectives as reflecting a failure to consider the interests of the Global South, but as a much more nuanced perspective with ethical priorities about preventing widespread pandemics and severe events with global repercussions.

Even if taken at face value, the argument assumes that the most important moral problem is that global health security does not include the concerns of the Global South. If there were other moral concerns, expanding global health security from a threat model to a wider reframing model would not solve these concerns, but simply mean the concerns were applicable to a wider range of areas. For example, if it was agreed that malaria was a global health security threat, an implication might be to focus on stopping the spread of mosquitoes rather than protecting people in existing areas with bed nets. Here, the moral problem would be about whether it is more important to focus on present persons suffering or future persons that might be affected (as is important in the threat model). This example shows that expanding global health security to further areas does not necessary mean that all moral tensions between the different models are solved.

A similar argument can be made for expanding global health security to non-communicable diseases, as found in the collective model. If global health security is problematic since infectious diseases cannot be addressed in isolation from the health systems these are managed in, or from the non-communicable diseases that affect a population’s resilience to infectious disease outbreaks, global health security should be expanded to include these areas [12,29–34,51]. Although this argument is primarily an empirical argument about whether it is effective to address infectious diseases without a wider systems approach, it may also have ethical connotations that it is *not fair* to try and manage or fund infectious diseases outbreaks in isolation to other components of health [46]. This is as it may prioritise the security aims of stopping diseases spreading at the expense of persons with ill health, who do not divide ill health by different aetiology of disease, but by those diseases that affect them whether this is COVID-19 or diabetes.

The obvious implication to expanding the model in these ways is that global health security essentially becomes equivalent in concept to global health. As already discussed in the introduction, this could have benefits if it brings global health to the attention of persons that otherwise would not be involved [9,19–21]. It also risks that securitising wider areas may not be convincing to policymakers, risking leading to the risk of oversecuritisation and ‘global health security fatigue’ [35,52]. However, the question about how ethically special an emergency is, and how this affects motivation to act are already addressed in the wider bioethics literature [53]. The unintended negative outcome here would be if attempts to widen the definition lead to weakening the credibility of advocates working on combatting epidemics and pandemics.

It would be premature to conclude from this analysis whether the scope of global health security should be expanded or not. However, it indicates that although calls for expanding the boundaries of global health security may be driven by moral concerns, care should be taken in considering whether global health security is the right solution to solving these moral concerns. This is because it is not clear that expanding the boundaries of global health security solves the key moral concern that global health security benefits the Global North at the expense of the Global South, or secondly, that the risks of expanding it from weakening the framing have been sufficiently considered. However, this does not exclude that there may be empirical reasons to expand, the strongest of which might be to do with the artificial divide between global health and global health security. However, this expansion would then need to be established using research methods beyond the scope of this ethical analysis.

It is also important to consider how political power and stability influence the choice of an appropriate model. The fact that interviewees may favour different models at different times does not necessarily mean they are able to advance the moral ideals embedded in those models, since such decisions are often driven by their organisation or nation. Moreover, the suitability of a model may depend on the context: for instance, the humanitarian model is often the most appropriate during a crisis, as it may be neither feasible nor desirable to promote the collective or reframing models in situations of severe political instability, whether caused by infectious disease, natural disaster, or conflict and violence.

### What this analysis adds

This analysis adds empirical evidence and nuance from those working in global health security, and considers implications of expanding the definition which have not previously been seen in the literature. Understandings of global health security among practitioners are multifaceted and complex and pull in different directions. For example, in some aspects their views might tend to pull in the direction of expanded accounts of global health security. In others they will tend to pull towards a narrower account. These tensions are something that those working in global health security have to struggle with as part of their roles. Consequently, these models offer important insights that may be useful to global health security professionals in understanding conflicting perspectives and navigating disagreements.

How global health security is defined carries significant practical consequences, shaping how normative frameworks and practitioner perspectives are translated into institutional arrangements, workforce development strategies, training programs, and broader system-strengthening efforts. For example, proponents of a threat-based model may adopt a relatively narrow conception of capacity building, prioritizing fields such as microbiology and epidemiology to enhance international preparedness, while giving less attention to systems-based approaches to regional health improvement. Such broader approaches might include investments in primary care infrastructure, governance mechanisms, or the accreditation of medical education [54,55]. These differences illustrate that ethical reasoning in global health cannot be confined to disciplinary silos; rather, they highlight the importance of an explicitly interdisciplinary perspective. Practitioners and policymakers in global health security should therefore reflect critically on how funds are prioritized, what is recognized as capacity building, how educational programs are structured, and who funds, participates in, and leads these initiatives.

The international relations literature criticises global health security as driven by self-interest and focused on the containment of diseases for the protection of Western countries [3,7,9]. This article provides a novel perspective by moving beyond these critiques to understand the enterprise as reflecting four distinct moral perspectives. Rather than being mischaracterised as a nationalist approach to global health security—one that asserts greater obligations to fellow nationals over non-citizens and emphasizes national self-interest [56] — we have shown through our analysis that the threat model is more accurately understood as a consequentialist approach, focused on minimizing mortality during epidemics and pandemics. The collective, re-framing, and humanitarian models—and to some extent the threat model—to some extent align with cosmopolitan perspectives on social justice, which place limited moral weight on national borders and view all individuals as having equal claims to aid. [57] This alignment stems from the fact that, although each model emphasizes different values, none rely on the importance of borders. The collective model focuses on cross-border cooperation to uphold individual health rights. The re-framing model advocates redistributing resources to support the most vulnerable. Both the humanitarian and threat models, while consequentialist in nature, prioritize alleviating present suffering and preventing large-scale outbreaks respectively, regardless of where they occur.

This highlights that individuals’ commitments to different models may reflect or be shaped by their broader ethical frameworks, including consequentialist and cosmopolitan perspectives. However, global health discourse is often framed in binary terms, such as casting global health security as the nationalist cousin of a more cosmopolitan vision of global health. These framings fail to capture the moral complexity and real-world dynamics of the field. More nuanced moral understandings move the debate beyond the simplistic binary by showing how diverse ethical perspectives can inform refined positions, shape policy discussions, and support the inclusion of a wider range of viewpoints.

## Limitations

One limitation of the study is that the majority of interviewees (74%, see Table 1) were working in high-income economies in the Global North. Although considerable effort was put in to recruit more individuals working in the Global South as the interviews progressed, it was noted that the perspectives of those working in the Global South did not, as a group, differ significantly from the Global North. Our hypothesis on why this was the case, was that many of these interviewees were embedded within this specific global context, attending the same international meetings and hearing the same discussions. The data collected also belies the global nature of interviewees. It was relatively common for interviewees to work in the Global South, or to mention they grew up in a Global South country, but worked for an international organisation or NGO with headquarters in the Global North. This level of detail was omitted to avoid interviewees being identifiable to their peers.

However, the absence of marked differences should not be taken to mean that positionality is irrelevant. Individuals working in contexts shaped by structural inequities may experience and interpret global health security priorities differently, even if these distinctions were not strongly articulated in our sample. It is possible that participation in a professionalized, internationally networked community attenuates more locally grounded moral frameworks. Future research that more deliberately centres actors operating outside national or international institutions may surface additional perspectives that refine or challenge the typology presented here.

There is also likely a bias towards those that agreed to interviews having an interest or openness to talking about ethics. Whilst there is not a way to fully address this bias, it is reassuring that there were negative perceptions and lack of understanding of what ethics is from interviewees as well as positive perceptions, indicating less social-desirability patterns from this professional group.

## Conclusion

This study provides one, if not the first, empirical accounts of how professionals view global health security. The analysis demonstrates that whilst the literature in global health security shows that this space has predominantly been described in terms of political institutions and international relations, one way it might be better described is as a complex moral landscape in which professionals navigate, whilst considering and challenging the moral boundaries of what global health security should include. These moral boundaries have not been previously acknowledged or analysed as moral problems, and therefore when this research analysed these as such, the assumptions, problems, and unanswered questions in these areas became apparent.

This article therefore provides a foundation for professionals and policymakers to engage more deeply with the ethical challenges embedded in global health security, encouraging more reflective and inclusive decision-making. A central takeaway is the urgent need to resist overly narrow and simplistic understandings and instead to embrace a more ethically aware and critically engaged approach to global health security. This is particularly important given the current challenges in global health security, from the withdrawal of the United States from WHO, [58] and the negotiations on the pathogen access and benefit-sharing system (PABS), which represent one of the most ethically complex elements of the Pandemic Accord. [59]

To move forward constructively, professionals and policymakers should use the four models as a foundation, and in some instances a tool, for integrating ethical analysis into policy development. They should engage with diverse perspectives, acknowledging that ‘global health security’ holds multiple different meanings and values. Used thoughtfully, the models can serve as both diagnostic and complementary tools to help identify which moral and operational perspectives are shaping decision-making, reveal potential gaps or tensions between actors’ priorities, and inform interventions that are ethically grounded, context-sensitive, and operationally feasible. Applied this way, the models support inclusive dialogue, help navigate disagreements, and strengthen consensus toward more effective and ethical global health security.
